# Guatemala paleoseismicity: from Late Classic Maya collapse to recent fault creep

**DOI:** 10.1038/srep36976

**Published:** 2016-11-15

**Authors:** Gilles Brocard, Flavio S. Anselmetti, Christian Teyssier

**Affiliations:** 1 School of Geosciences, University of Sydney, NSW 2006, Australia; 2 Institute of Geological Sciences and Oeschger Centre for Climate Change Research, University of Bern, Baltzerstrasse 1-3, CH-3012 Bern, Switzerland; 3 Department of Earth Sciences, University of Minnesota, Minneapolis MN 55455, USA

## Abstract

We combine ‘on-fault’ trench observations of slip on the Polochic fault (North America-Caribbean plate boundary) with a 1200 years-long ‘near-fault’ record of seismo-turbidite generation in a lake located within 2 km of the fault. The lake record indicates that, over the past 12 centuries, 10 earthquakes reaching ground-shaking intensities ≥ VI generated seismo-turbidites in the lake. Seismic activity was highly unevenly distributed over time and noticeably includes a cluster of earthquakes spread over a century at the end of the Classic Maya period. This cluster may have contributed to the piecemeal collapse of the Classic Maya civilization in this wet, mountainous southern part of the Maya realm. On-fault observations within 7 km of the lake show that soils formed between 1665 and 1813 CE were displaced by the Polochic fault during a long period of seismic quiescence, from 1450 to 1976 CE. Displacement on the Polochic fault during at least the last 480 years included a component of slip that was aseismic, or associated with very light seismicity (magnitude <5 earthquakes). Seismicity of the plate boundary is therefore either non-cyclic, or dominated by long-period cycles (>1 ky) punctuated by destructive earthquake clusters.

The North America-Caribbean plate boundary is a left-lateral, mostly submarine structure that surfaces here and there through the Greater Antilles and Central America. The most recent major destructive earthquake on the plate boundary occurred in Haiti in 2010 CE, as a M_w_ 7.0 event that claimed more than 220,000 lives and caused financial damage reaching 100% of the national gross domestic product (Bolton^1^; Calais^2^). In Guatemala, the last major destructive earthquake reached M_w_ 7.5 in 1976 CE, claimed 23,000 lives, and left 1.5 million people homeless (Olcese *et al*.^3^). In Guatemala the North American-Caribbean plate is regarded as the main source of low-recurrence, high-intensity earthquakes for most cities, including Guatemala City^4^. In spite of its large hazard potential, the seismic history of the boundary is poorly known and relies on the compilation of written reports of earthquake-related destruction (White^5^), and on the observation of earthquake-induced damages in pre-Columbian settlements (Kovach^6^). Here we combine an *on-fault*^7^ study of recent fault slip with a *near-fault* record of ground shaking-induced turbidites in a lake (seismo-turbidites or seismites). Combining both methods informs the paleoseismic behavior of the plate boundary over a millennial timescale. On-fault trenches provide direct evidence for fault displacement, but generally do not allow the distinction between seismic and aseismic slip. In paleoseismological studies ground disruptions are almost invariably attributed to seismic slip^8^ in spite of growing evidence that macroseismic displacements accommodate only a fraction of the total strain that accumulates in the upper crust across plate boundaries (Fröhlig and Wetzel^9^). Moreover, post-rupture creep (afterslip)^10^, creep at shallow depth above a locked seismogenic zone^11^,^12^,^13^, and generalized creep^14^ are commonly measured on many faults today. Therefore, the nearly systematic assignment of surface dislocations to earthquakes may lead to an overestimation of past seismic activity. Detection of past creep is difficult, however, and is based on the lack of features typically produced by rapid, seismic surface ruptures, such as deformation bands and broken grains in loose surface sediments^15^, or the generation of fault scarps and associated rapidly-grown colluvial wedges, especially in areas undergoing rapid sedimentation^16^. Here instead we use a near-fault record of lacustrine seismites to test whether disruptions to floodplain soils produced by a major fault were accompanied by ground shaking. We show that some of the fault slip observed across the floodplain occurred during a 5-century-long period of tectonic quiescence, during which no seismites were generated. We interpret this as an episode of aseismic or low-intensity seismic creep. This period of quiescence was preceded by a cluster of earthquakes that generated closely spaced seismites at the end of the Maya Classic Period, which damaged several Maya cities. We explore the implications of this record for the seismic behavior of the plate boundary, the demise of the Classic Maya civilization, and the likelihood of future major earthquakes.

## Setting

**Figure 1:**
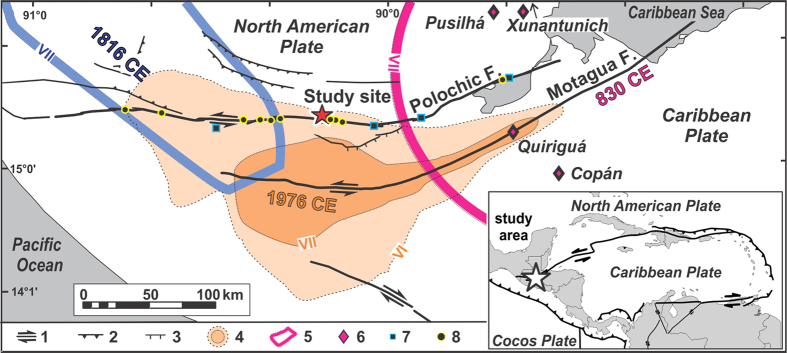
Structure of the North American-Caribbean plate boundary in Guatemala. Faults: 1: strike-slip, 2: reverse, 3: normal. 4 and 5: major earthquakes, isoseismal intensities in roman numbers, 4: 1976 CE^18^, 5: 1816^19^, modified, and 830 CE^6^, 6: cities damaged around 830 CE, 7: hot springs, 8: sulfate-rich springs. Inset: location of the study area along the Caribbean-North American plate boundary. Map generated using ArcGIS 10 and CordelDraw 12 (www.coreldraw.com).

**Figure 2:**
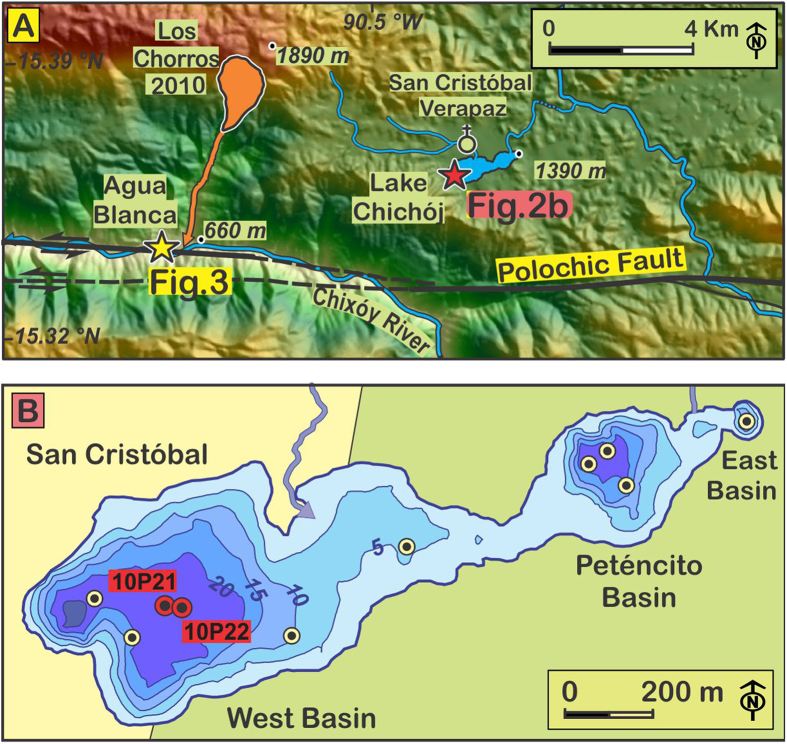
Location of on-fault and near-fault observations. (**A**) Location of the trenches (yellow star) and lake (red star) with respect to the Polochic fault trace (black solid lines). Orange: 2009 CE Los Chorros avalanche scar, deposits, and debris flow corridor. (**B**) Close up map of Lake Chichój with coring sites (dotted circles). Core used for the composite section in red, cores in yellow not used in this study but described in Fig. S2-1). Map generated using ArcGIS 10 (https://www.arcgis.com) and CorelDraw 12 (www.coreldraw.com).

In Guatemala, two major structures, the Motagua and Polochic faults (Fig. 1) accommodate under the current interseismic cycle roughly 3/4 and 1/4 of the ~2 cm/y left-lateral motion between the North American and Caribbean plates^17^. The Motagua fault ruptured over 250 km during a M_s_ 7.5 earthquake in 1976 CE^18^. No other major earthquake can be ascribed with certainty to any of the faults of this plate boundary in Guatemala, in the absence of direct geologic evidence. The distribution of earthquake-induced damages in Classic Maya cities however suggests that another major earthquake occurred around 830 CE^6^ on the Motagua fault (Fig. 1). The spatial spread of reported earthquake-related destructions after the Spanish colonization does not evidence any other major earthquake on the Motagua fault; however the reports suggest the possibility of three major earthquakes in 1538, 1785, and 1816 CE on the Polochic fault^5^,^19^. It has been stressed that the collective inventory of earthquakes along the Polochic and Motagua faults over the last millennium seems insufficient to accommodate the total left-lateral motion accumulated across the plate boundary over the period, suggesting a deficit of major seismic events^20^. Over the 20^th^ century however, the instrumental record of M < 5.5 earthquakes does not display a severe deficit^21^,^22^,^23^. Direct geological observations of displacements across the plate boundary are thus far restricted to observations in 10–17 ky-old alluvium along the Polochic fault^24^. Field evidence for more recent motion is lacking on both faults. In this study we combine on-fault observations of surface displacements in soils deposited during the past millennium over the Polochic fault trace at Agua Blanca, with a nearby record of lacustrine seismites that spans the past twelve centuries in lake Chichój (Fig. 2).

## Results

### Age of the latest surface rupture on the Polochic fault

**Figure 3:**
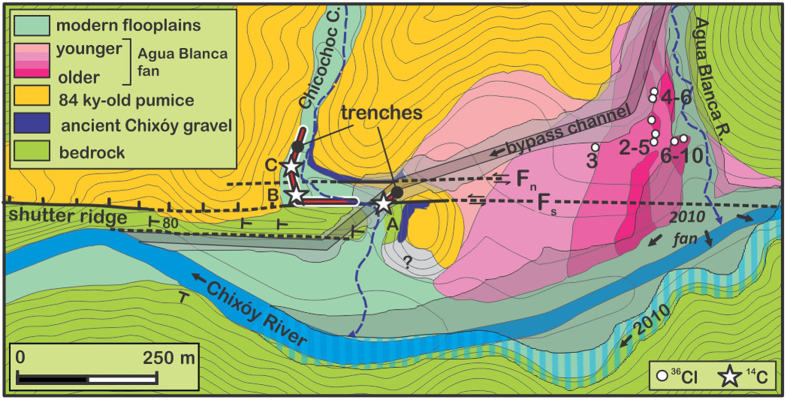
Geological map of the Agua Blanca area with location of trenches (red lines). Topographic contour line spacing: 10 m. Grey-shaded areas: footprint of post-2010 debris flows and earthworks. Note the shift of the Chixoy River from its pre-2010 bed (dark blue) to its corrected bed (striped light blue). White dots: ^36^Cl surface exposure dating^24^. White stars: ^14^C soil dating. Map generated using ArcGIS 10 (https://www.arcgis.com) and CorelDraw 12 (www.coreldraw.com).

**Figure 4:**
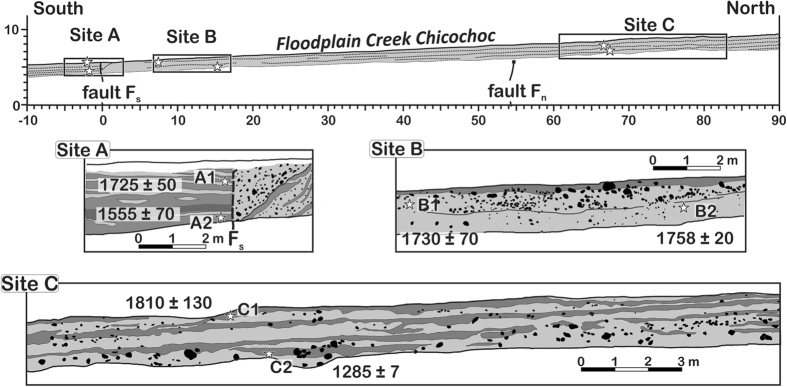
Cross sections in the floodplain of Creek Chicochoc, showing the overall floodplain architecture, and the relationships between paleosols (dark gray), fault strands (F), and ^14^C-dated samples (white stars, A1–C2) with calendar ages reported at 68% confidence level (Table S1, additional information). Light gray: sand and gravel, with largest gravels in black.

The floodplain of Chicochoc Creek in Agua Blanca (Fig. 3) exposes a regular alternation of paleosols and sandy-gravelly alluvium layers, crosscut by the active trace of the Polochic fault (Fig. 4). At this specific site the Polochic fault is defined by one single vertical plane that directly juxtaposes tilted gravel alluvium to stacked paleosols (Fig. 4 and S1-1). Juxtaposition of the gravels and soils requires at least a meter-scale slip on the fault plane. Destruction of the topsoil prevented determining whether the trace disrupts the ground surface. However, observations conducted at the site before the opening of the trenches failed to detect any topographic discontinuity^24^, suggesting that the fault zone used to be sealed by the topsoil. ^14^C dating indicates that sedimentation has been fairly uniform over the past 5–7 centuries throughout the floodplain, at a rate of 2–4 mm/y (Additional information, Table S1-2). The youngest dated displaced soil developed between 1665 and 1813 CE. The last displacement on the fault therefore occurred after 1665 CE, and possibly after 1813 CE.

### Seismite succession in Lake Chichój

**Figure 5:**
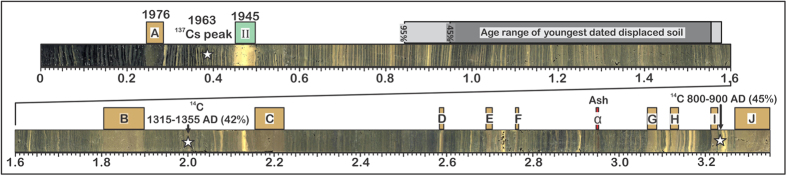
Composite section from the west basin of Lake Chichój. Layers A–J are seismo-turbidites (light brown marks); layer II (turquoise) is a flood layer (1945 CE) and layer *α* is a mafic ash fall. Grey-shaded area: depth range corresponding, within uncertainties (one sigma dark grey; two-sigma light grey), to the age of the youngest ^14^C-dated displaced soil at Agua Blanca. It is drawn to show where seismic-related disruptions should be observed, had slip produced ground shaking > MMI VI. White stars indicate age markers (^137^Cs peak and calibrated radiocarbon ages).

**Figure 6:**
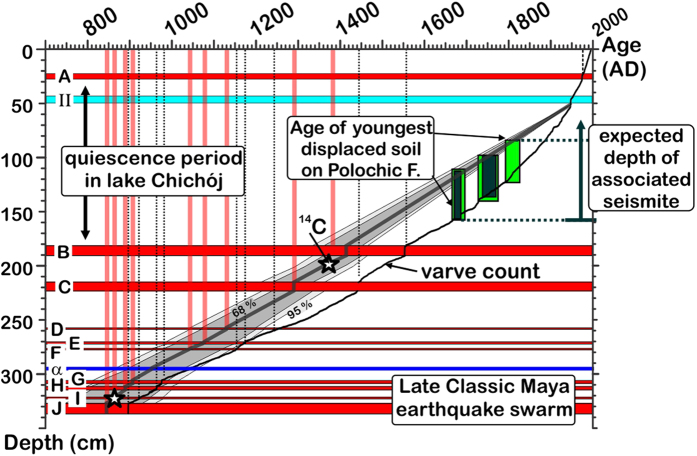
Seismo-turbidite depths (red horizontal bars, A–J), and inferred calendar ages according to the ^14^C dating (red) and varve counting models (black). Green bars mark ^14^C age range of the most recent dated displacements on the Polochic fault in Agua Blanca, and corresponding depth range where tectonic disruptions should have been recorded, had this displacement generated earthquakes reaching MMI VI and higher at the lake.

The 3.3 m-long sedimentary record of Lake Chichój includes 10 dark-colored seismoturbidites (*A* to *J*, Fig. 5). Radiocarbon and varve-based models are in good agreement both at the top and base of the section (Fig. 6), however they reach an age separation of up to 100 years half way up section. This discrepancy may arise from the incompleteness of the varve counting, or from deposition of the wooden material in the lake decades after its growth. We therefore regard the two age models as upper and lower estimates of the seismite ages. Notwithstanding both age models indicate that 10 earthquakes reaching MMI ≥ VI occurred during the past 12 centuries, separated by very irregular return intervals.

## Discussion

### Earthquake cluster at the end of the Maya Classic Period

Lake Chichój experienced a long period of earthquake quiescence from the 16^th^ century to the devastating 1976 CE Motagua earthquake (layer *A*). Five earthquakes (layers *B–F*) occurred during the Maya Post-classic period (~1550-1000 CE), and a cluster of four earthquakes (layers *G–J*) occurred in less than a century at the end of the Maya Classic period (750–900 CE). The oldest seismoturbidite coincides, within age-model accuracy, with an archeologically defined major earthquake in 830 CE which, from the areal distribution of destructions (Fig. 1), is believed to have occurred on the Motagua fault^6^. This cluster occurred during the widely-debated collapse of the Classic Maya civilization, a period characterized by massive depopulation of the Petén-Yucatán lowlands farther north, and the abandonment of its largest urban centers^25^. The origin of the collapse is multi-facetted and is thought to have societal and natural causes, with climate change (i.e. droughts) and soil erosion as possible important triggers, or contributing factors^26^,^27^,^28^.

Growing anthropogenic pressure on the environment and its natural resources is believed to have reduced societal resilience to environmental stress, in particular the ability to cope with droughts^29^. Yet, droughts are mostly a challenge for settlements located in the karstic Petén-Yucatán lowlands. They are much less of a concern in the wet tropical mountains that surround the Motagua and Polochic faults. Yet, Maya cities in Central Guatemala and Honduras were also widely affected by the collapse. Earthquake-induced destructions have been found in several large Classic Maya cities located near the plate boundary (Fig. 1), some of which were not reconstructed afterwards^6^, and the age of these destructions coincides with the timing of the seismite cluster found in Lake Chichój. The seismite cluster therefore suggests that the Classic Maya collapse occurred during a period of damaging seismic unrest. Unrest consisted either in one major and several smaller earthquakes only reaching high intensities locally near the lake, or in a series of several major earthquakes (four events between 840 and 910 CE according to our proposed dating scheme). A single earthquake affecting a resilience-impaired society is sufficient to trigger institutional collapse, as illustrated, along the same plate boundary, by the 2010 earthquake in Haiti^1^,^30^. The repetition of seismic destructions, even on more resilient societies, can motivate city abandonment or relocation, as illustrated in Guatemala by the relocation of the capital city Antigua Guatemala during the 18^th^ century after a series of shallow destructive earthquakes affecting the Central American volcanic arc^31^.

### Creep on the Polochic Fault

An upper crustal earthquake of M ≥ 5 occurring within 7 km of Lake Chichój on the Polochic fault is likely to be felt with MMI ≥ VI at the lake. As a result, any decimeter-scale seismic rupture at the ground surface in Agua Blanca is expected to leave a detectable seismic imprint in the sediment record of Lake Chichój. The meter-scale fault displacement observed at the trench site did not generate any associated seismic disruptions in Lake Chichój, suggesting that fault slip occurred either aseismically or through the repetition of small earthquakes. The earthquake-recording threshold of MMI VI established in Lake Chichój for the 20^th^ century^32^ is similar to that of many other lakes worldwide^33^,^34^, although higher thresholds of MMI VII have been documented^35^. The quiescence of the lake record over the past 5 centuries (ending in 1976 CE) could have several explanations: (1) associated sedimentary structures have been missed during coring, (2) the earthquake-recording sensitivity of the lake was lowered significantly over this time interval, or (3) earthquakes had different source characteristics. (1) The small size of the lake, dense core array, and general spreading of the turbidites across sub-basin floors makes it highly unlikely that a seismoturbidite could have been missed (2) Deposition of closely spaced seismites at the base of the composite section indicates that sedimentation rate has been sufficiently high to rapidly recharge slopes, keeping the lake system susceptible to the next earthquake-triggered slope failure. In addition, the sub-basin from which the composite section was retrieved did not experience alterations of its topography, sediment composition, or sedimentation rate such as to modify its recording sensitivity to the point where the distant 1976 CE earthquake could generate extensive turbidites and slumps, while high-intensity earthquakes occurring within 7 km of the lake would not leave any detectable imprint. (3) Slope failure is sensitive to the spectral distributions of seismic waves^36^, and fault rupture propagating close to, or at, supershear velocities tend to trigger more damage than other earthquakes. Putative differences in seismic source characteristics from an earthquake to the other, however, cannot distort the paleoseismic record to the point where major earthquakes occurring next to the lake could be so much less damaging than the distant 1976 CE earthquake. We therefore conclude that the lack of seismite in Lake Chichój does reflect a period of seismic quiescence, during which slip on the Polochic fault was produced by permanent or intermittent creep, generating microseismicity or/and relatively small earthquakes (M < 5).

Seismic creep on the Polochic and Motagua faults may account for the large earthquake deficit observed on this plate boundary^20^. It is not unexpected on the Motagua fault, where large amount of afterslip were observed following the 1976 CE earthquake^10^. The fault runs along a oceanic suture delineated by serpentinite mélanges, which are thought to be directly or indirectly conducive to creep^37^. No such mélanges are present along the Polochic fault. However, the noticeable abundance of hot springs dotting the fault (Fig. 1) suggests the existence of high pore pressure at depth, which favours creep^38^. Gypsum and dolomite are present in large amounts throughout the Cretaceous carbonates^39^ which flank the northern side of the Polochic fault over most of its length in Central Guatemala. Although often poorly exposed at the surface, gypsum and dolomite feed numerous sulfate-rich springs (Fig. 1). They are encountered at shallow depth, and crop out most extensively near the site of trenching^22^, where they may therefore promote aseismic creep^40^,^41^.

### Seismic hazard on the Polochic fault

The temporal seismic pattern found in this study differs from earlier estimates based on historical archives. In particular we did not find evidence for major earthquakes thought to have occurred in 1816, 1785, and 1538 CE^42^. Amongst these, only the 1816 CE can safely be regarded as a major earthquake, based on the areal extent of the damages^43^. However, its attribution to the Polochic fault has been questioned, and so was the areal extent of its associated destructions, which might be better explained by a succession of smaller events on several faults located farther west^44^. Less than a handful of archives mention destructions around Lake Chichój, at the eastern termination of the inferred rupture area, and these are vague or dubious (Supplementary Information S3). We therefore consider the geological record more reliable there, with a five-century long gap of seismicity following a period of elevated seismicity. The creeping behaviour observed near Lake Chichój could be episodic, and increase in association with large seismic ruptures farther away along the plate boundary^13^, such as for example the 1816 CE earthquake. The plate boundary does not exhibit a cyclic return of earthquakes over the duration of our observation window, but a cyclic pattern cannot be excluded. It would then be characterized by a long return period (>1.5 ky). As a result, the probability of the next large earthquake on the plate boundary cannot be assessed based on the present record. Some hypotheses can be made, however, regarding the potential maximal magnitude of a major earthquake on the Polochic fault, depending on the downward extent of creep along the fault plane. If creep occurs throughout the fault plane, it entirely accommodates fault motion, and no potential seismic slip is currently building up along the Polochic fault. Measurements on well-instrumented continental strike-slip faults find that wherever creep occurs, it remains restricted to the upper crust, above a deeper zone of either slow earthquakes and tectonic tremors^45^,^46^, or above a locked, seismogenic zone that ruptures either in M4-5 earthquakes^12^, or in infrequent M6-7 earthquakes^11^. The Polochic fault currently produces micro-seismicity to a depth of 10 km and M4.5–5.5 earthquakes between 5 and 10 km^23^, suggesting the existence of a locked zone at depth. There is also geological evidence for past episodic, possibly seismogenic deformation at the surface^24^. We therefore consider unlikely that fault creep also occurs at depth. If nonetheless generalized creep occurs at the studied site, then the alternation, over the course of several centuries, of creep and earthquake swarms (that is, of fault-strengthening and fault-weakening slip phases respectively) could perhaps result from multi-centennial variations in fluid pore pressure, due for example to large stress fluctuations on the Polochic fault following large earthquakes on the Motagua fault^5^, or from multi-centennial variations in precipitations^12^,^13^, with droughts being conducive to earthquakes. Fault aging is thought to affect the duration of the interseismic period, and may evolve with fault development in such a way as to change the long-term slip behaviour of a fault. However such change is generally envisioned as unidirectional^47^, rather than switching back and forth over the course of several centuries.

We assess the maximum seismic hazard on the Polochic fault by assuming that the fault possesses a fully locked zone at depth capable of generating M6-7 earthquakes. The lack of seismite implies that no such earthquake has occurred on the Polochic fault during at least the past 480 years, since the generation of turbidite *B*. Afterslip (creep relaxation during the months following a seismic rupture) alone cannot account for the slip documented at Agua Blanca. Indeed, the earthquake related to turbidite *B* occurred before 1557 CE, whereas displacements at Agua Blanca continued after 1680 ± 15 CE. Using the current <5 mm/y interseismic fault velocity^17^, the time-averaged fault velocity of 2.9 ± 0.4 over the past 7 ky^32^, and the time-average fault velocity of 1.3 mm/y over the past 84 ky^24^, we find that the locked zone of the fault has accumulated between 0.6 m and 2.2 m of potential slip since deposition of seismite *B*. If such potential slip were released during a single rupture event, it would generate a major earthquake. Accumulated strain could be even larger, because turbidite *B* and some of the earlier turbidites may also have been generated on regional faults other than the Polochic fault^5^,^6^,^44^.

## Methods

### Trenches on the Polochic fault

The Polochic fault was trenched at a site of rapid ongoing sedimentation, where two mountain streams joins the large Chixóy River, which in this area flows along the fault trace^32^ (Fig. 3). One of the two streams, Agua Blanca creek, has built a large alluvial fan across the Polochic fault. The fan is made of successive debris flows. Cosmogenic ^36^Cl exposure dating of boulders scattered over the fan surface has revealed four pulses of fan growth during the Holocene^24^. A fifth pulse started in 2009 CE following a rock avalanche in the river catchment (Fig. 2A). The rock avalanche deposit has since been eroding away, feeding numerous debris flows that progressively dammed the Chixóy River, urging the construction of a bypass channel and of service roads aimed at mitigating the impoundment (Fig. 3 and Supplementary Fig. S1-1). The resulting earthworks have exposed soils recently displaced by the Polochic fault. We studied and sampled these soils along trenches excavated in 2009 and 2012 across the finer-grained and well-stratified floodplain deposits of Chicochoc Creek, a seasonal stream flowing through Agua Blanca. The trenches provided continuous exposure perpendicular to the Polochic fault, over a projected distance >100 m (Fig. 4, Additional Information Fig. S1-1). Seven pieces of wood collected 75, 10, and 0 m north of the active strand of the fault were radiocarbon-dated at the National Ocean Sciences Accelerator Mass Spectrometry (NOSAMS) facility at Woods Hole Oceanographic Institution, Massachusetts (Additional Information, Table S1-2). Radiocarbon ages were converted to calendar ages using software Bcal (http://bcal.sheffield.ac.uk)^48^.

### Sediment coring in Lake Chichój

Lake Chichój, located 7 km east of the trenches, lies within 2 km of the Polochic fault (Fig. 2). It is also located 50 km north of the Motagua fault (Fig. 1). Fifteen sediment cores were recovered from three lake sub-basins in 2009 and 2010 using a 63-mm-diameter UWITEC hammer corer (Fig. 2B, and Supplementary Fig. S2-1). Cores were opened and photographed at the Swiss Federal Institute of Aquatic Science and Technology (Eawag) in Duebendorf, Switzerland. Lake sediments are dominantly composed of silty clays, with dark- and light-colored laminae produced by the annual alternation of dry and wet seasons (true varves), interlayered with turbidites and mass-movement deposits^22^.

Flood-related and earthquake-related turbidites are very distinct in lake Chichój. Flood-induced turbidites are light-colored and rich in pumice, magnetite, and quartz, whereas earthquake-induced turbidites (seismites) remobilize dominantly shallow lacustrine sediment which contains organic debris and biochemically precipitated carbonate^22^. Comparison of 20^th^ century seismites and of isoseismal maps of damaging 20^th^ century earthquakes shows that seismite generation in Lake Chichój occurs at ground-shaking Mercalli Modified Intensities (MMI) ≥ VI^22^.

A 3.3 m-long continuous composite sedimentary section was constructed using the two longest cores (Fig. 5) retrieved from the West Basin. The age model of the composite section is based on three independent datasets. (1) a ^137^Cs-^210^Pb inventory performed on the topmost 70 cm of the section constrains the age of sediments deposited during the 20^th^ century^22^. (2) Varve counting down-core starting at turbidite *A* (1976 CE earthquake) provides precise age estimates down to ~1820 CE. Farther down core, the layering is less contrasted and finer, which makes the counting less precise. Varve-derived ages are therefore regarded as minimum ages. (3) AMS-^14^C-dating of wooden debris was performed at the ETH Zurich, Switzerland. Ages are δ^13^C-corrected based on the ^14^C concentration in each sample. Seismoturbidite ages (Supplementary information, Table S2-2) were calculated by linear interpolation of the sedimentation rate between ^14^C-dated samples (after removing the rapidly-deposited turbidites); then by linear interpolation between the shallowest ^14^C sample and 1945 CE flood layer II^22^, and, below the deepest dated sample, by linear propagation of the deepest integrated rate.

## Additional Information

**How to cite this article**: Brocard G. *et al*. Guatemala paleoseismicity: from Late Classic Maya collapse to recent fault creep. *Sci. Rep.* **6**, 36976; doi: 10.1038/srep36976 (2016).

**Publisher’s note**: Springer Nature remains neutral with regard to jurisdictional claims in published maps and institutional affiliations.

## Supplementary Material

Supplementary Information
