# Supplementary material for: Guatemala paleoseismicity: from Late Classic Maya collapse to recent fault creep
Source: Sci Rep. 2016 Nov 15;6:36976. doi: 10.1038/srep36976 (PMC5109539; doi:10.1038/srep36976)
Supplement: Supplementary Information [file srep36976-s1.docx]

**Nature Scientific Reports**

**Supplementary information to**

**Guatemala paleoseismicity: from Late Classic Maya collapse to recent fault creep**

By Gilles Brocard, Flavio Ansemetti and Christian Teyssier

**S1- Agua Blanca: detailed tectonic description**

The Polochic fault exhibits a well-constrained total displacement of 125 km^1^, which initiated between the Eocene and the Upper Miocene ^1-3^. In Agua Blanca, a major strand of the fault crosses an area of rapid sedimentation, along the right bank of the Chixóy River (Fig. 3). Earthworks for a bypass channel have revealed the internal structure of the Chicochoc floodplain, exposing its recent soils, as well as the internal structure of tectonically-displaced ridges between the Chicochoc and Agua Blanca creeks. A trench excavated in 2010 through the modern floodplain of Chicochoc Creek provided continuous exposure from upstream of fault strand *Fn* down to immediately upstream of fault strand *Fs*. *Fs* was exposed along the banks of the bypass channel in 2012.

The shutter ridge south of *Fs* is made of a core of vertically-bedded Permian carbonate, overlain by perched alluvial gravel from the Chixóy River, easily fingerprinted by its content in serpentinite clasts. Similar gravel is found at the level of the Chicochoc floodplain north of *Fn*, indicating that the shutter ridge has grown through a combination of left-lateral faulting and vertical uplift. The hills surrounding Agua Blanca are made of rhyolitic pumiceous ash-flow deposits of the Los Chocoyos formation, deposited during a Volcanic Explosive Index (VEI) 7 eruption of the Atitlán Caldera ^4^ some 84 ky ago ^5^. During this event the site of Agua Blanca was buried into ~100 m of pumice, and the topography has since been incised into these deposits. The 84 ky-old resurfacing provides an upper age limit for the development of the left-lateral deflection of the Chicochoc creek, and for the shearing of the divide between the Chicochoc and Agua Blanca creeks. This deflection is used to infer a minimum left-lateral fault velocity of 1.3 mm/y over the past 84 ky in Agua Blanca ^2^.

A single fault plane *Fs* currently crosscuts the Chicochoc floodplain. However, the ridge between Agua Blanca and Chicochoc Creeks exposes two parallel planes a few tens of meters apart (*Fn* and *Fs*, Fig.3, 4 and S1-1). *Fn* dips 50-60° to the south and exhibits three generations of slickensides. The two older generations only affect the ancient alluvium of the Chixóy River, which predates the 84 ky-old pumice. They show a change from pure left-lateral slip, to a 25% normal component. The third generation of slickensides, imprinted into both the alluvium and the 84 ky-old pumice, documents an increase of transtension with a 50% normal component. This fault plane does not crosscut the recent soils of the Chicochoc floodplain (Fig.4). *Fs* is a vertical plane that crosscut the recent soils of the floodplains (Fig. S1-1/3). It also crops out in the ridge between the Chicochoc and Agua Blanc ridge (Fig. S1-1/1). Minor fault planes dipping 70-80° N immediately south of *Fs* display pure normal motion; their footwall is made of ancient Chixóy River gravel and their hanging wall of 84 ky-old pumice. This general arrangement indicates that the Polochic fault in Agua Blanca is a small negative flower structure with strong partitioning of deformation at the surface, including purely left-lateral active faulting on the strand crossing the floodplain of Creek Chicochoc in the center, and parallel fault planes displaying various amounts of transtension north and south of the main fault surface. Radiocarbon dating of the floodplain (Table S1-2) indicates that only *Fs* has been active during the past millennium.


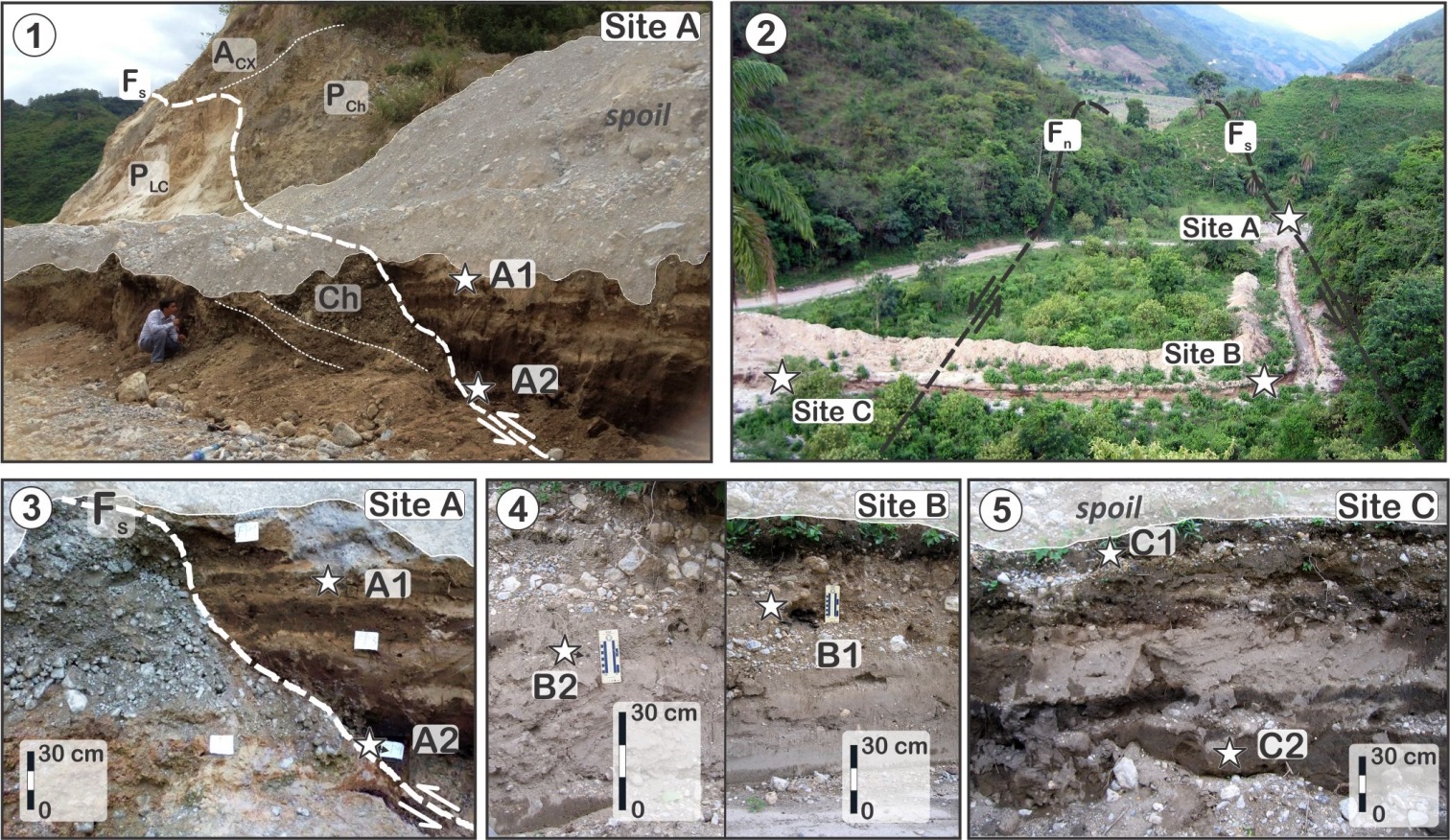


**Figure S1-1**. Field images of outcrops and sampling along Creek Chicochoc. 1) overview of the study site, 2) soils displaced by *Fs*: Acx: uplifted gravel of the Chixóy River, Ch: gravelly channel deposited by Creek Chicochoc, P_ch_: Permian Chochál carbonate, P_LC_: 84 ky-old Los Chocoyos pumice, panel 3), 4) and 5): close-up views of the soils dated at sites A, B, and C, respectively.

Radiocarbon dating

Seven wood samples were selected for ^14^C dating, at three sites along the floodplain of Creek Chichococ (Fig. 4, S1-1, Table S1-2).

| Sample | Depth | ^14^C age range | NOSAMS ID # | ^14^C age probability peaks  BCal (95%) | ^14^C age probability peaks BCal (68%) |
| --- | --- | --- | --- | --- | --- |
|  | *cm* | *Years (BP)* |  | *Years (cal AD)* | *Years (cal AD)* |
| **A1** | 50 | 165 ± 20 | 118160 | (1680 ± 15) (1755 ± 30) (1804 ± 9) | (1677 ± 7) (1756 ± 21) |
| **A2** | 140 | 365 ± 20 | 118161 | (1493 ± 31) (1595 ± 38) | (1501 ±17) (1610 ± 18) |
| **B1** | 45 | 160 ± 20 | 118157 | (1681 ± 15) (1755 ± 30) (1805 ± 9) | (1664 ± 6) (1788 ± 10) |
| **B2** | 85 | 210 ± 20 | 118158 | (1665 ± 15) (1746 ± 8) (1782 ± 20) | (1758 ± 20) |
| **C1** | 65 | 155 ± 20 | 118156 | (1682 ± 14) (1754 ± 28) (1806 ± 9)  (1842 ± 6) (1860 ± 9) (1934 ± 7) | (1682 ± 7) (1749 ± 19)  (1934 ± 7) |
| **C2** | 160 | 695 ± 20 | 118155 | (1285 ± 14) (1375 ± 6) | (1285 ± 7) |

Table S1-2. Radiocarbon ages from samples collected in the floodplain of Chicochoc Creek.

**S2. Laguna Chichój - Overall results of the coring campaign**

Fifteen cores were retrieved in 2010 from three basins in Lake Chichój (Fig. S2-1). The cores retrieved from basins slopes and on the basin floors near the base of the surrounding slopes intersected abundant slumps and were not therefore suitable for constructing a long-term chronology. Varves deposited above the slumps indicate that most of the intersected mass movements were triggered by the 1976 CE earthquake on the Motagua fault. Mass wasting was then so extensive that slumped material completely covered the floor of the Central (Peténcito) sub-basin. Cores retrieved from shallow sills and upper slope locations do not contain turbidites, due the fact that turbidites in lake Chichój have been deposited by low-energy density current that have remained confined within the deepest parts of the basins. Only the cores retrieved from the central part of the west sub-basin retain undisturbed successions, and these were used to construct the composite section.

It was assumed that all dark turbidites correspond to mass wasting events in the lake triggered by earthquakes, due to the perfect correspondence of earthquakes and dark turbidites in the 20^th^ century ^6^. The fact that dark-colored turbidites and slumps in the 20^th^ century were all earthquake-related suggests that ground shaking is sufficiently frequent to purge the lake from all gravitational instabilities that would otherwise develop during inter-seismic periods. Synchronous mass movements in the various sub-basins during earthquakes are well observed in the 20^th^ century. Such criterion could not be used for the analyze former earthquakes due to the exceedingly widespread disruptions resulting from the 1976 CE earthquake in the Peténcito Basin, which prevented reaching older levels.


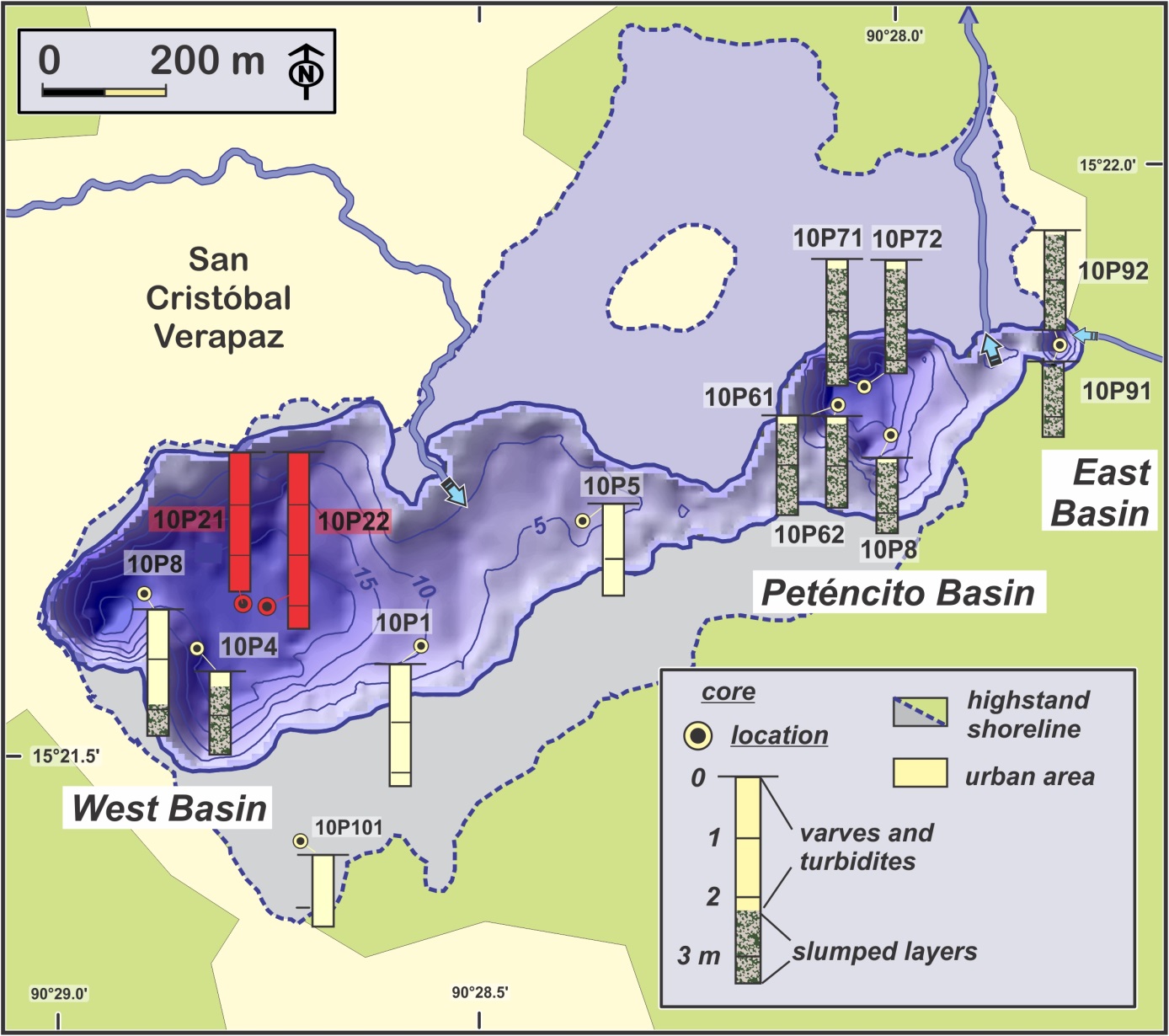


Figure S2-1. Location of cores retrieved from Lake Chichój. Red: cores yielding a continuous record over the past millennium. Yellow: other cores. Bathymetric contour spacing: 5m^6^.

Map generated using ArcGIS 10 (https://www.arcgis.com).

**Radiocarbon dating of the seismoturbidites**

| Layer | Age Varve counting | ^14^C age range | ETHZ Lab ID | ^14^C age model (95%) | ^14^C age  Model (68%) |
| --- | --- | --- | --- | --- | --- |
|  | Years (AD) | Years (BP) | # | Years (cal AD) | |
| **B** | 1557 |  |  | (1413 ± 56) | (1414 ± 42) |
| *10P21-1* |  | *571 ± 38* | *49535* | *1363 ± 64* | *1364 ± 48* |
| **C** | 1444 |  |  | (1287 ± 68) | (1289 ± 50) |
| **D** | 1242 |  |  | (1128 ± 76) | (1134 ± 59) |
| **E** | 1172 |  |  | (1081 ± 79) | (1087 ± 61) |
| **F** | 1155 |  |  | (1044 ± 81) | (1050 ± 62) |
| **α** | 1044 |  |  | (959 ± 85) | (965 ± 63) |
| **G** | 980 |  |  | (912 ± 88) | (918 ± 65) |
| **H** | 966 |  |  | (893 ± 89) | (900 ± 66) |
| **I** | 921 |  |  | (870 ± 90) | (877 ± 67) |
| *10P21-2* |  | *1,160 ± 35* | *42293* | *870 ± 90* | *877 ± 67* |
| **J** | 896 |  |  | (851 ± 91) | (858 ±67) |

Table S2-2. Age of earthquake-induced turbidites in Lake Chichój, according to varve counting and ^14^C dating.

**S3. Comparison to historically-reported earthquakes**

We found no trace of the major earthquakes of 1785 CE and 1816 CE, ascribed to the Polochic fault on the basis of building damage zoning ^7,8^. Proposed reconstructed isoseismal maps imply that the lake area would experience MMI > VII during these events and should therefore clearly show signs of ground shaking. Inspection of the cores failed to show any seismite over the corresponding depth interval (Fig. 5). The only observed perturbation is a few-cm-thick duplex-shaped deformation in core 10P21 during the early 20^th^ century, and possibly another one in early 19^th^ century. However corresponding deformations structures are not found in the two parallel cores retrieved at the same site (09P2 and 10P22). For this reason, and because the 19^th^ century perturbation is located near the level at which core 10P21 was sliced, we interpret these disturbances are cutting/coring artifacts. This is also supported by the fact that there is no reported earthquake reaching an MMI ≥ VI during the early 20^th^ century ^6^. In addition, if a major earthquake had occurred on the Polochic fault at these times, it would be expected to produce disturbances equal or greater than the widespread landslides and turbidites produced by the much more distant 1976 CE earthquake. The 1816 CE earthquake is the only major earthquake for which a substantial body of reports exists ^8^. The extent of the damage zone in Central Guatemala and the lake area, however, only relies on two reports: a report in Guatemala City, the wording of which is ambiguous regarding destructions in Central Guatemala; and a local report from San Cristóbal Verapaz, documenting earthquake-related damage to the church of Santa Cruz, 5 km east of the lake, two years after the event, while not damage is reported to the church of San Cristóbal at the time of the earthquake. Because most of the reported destruction occurred farther west in Guatemala and in Chiapas, Mexico, the 1816 CE earthquake has also been explained as a swarm of events occurring within a few days or a few hours on faults located in these regions ^9^.

The lake itself has been described as the product of earthquake activity. Reports indicate that the lake formed by catastrophic ground collapse, possibly following an earthquake in 1590 CE ^10^. The extent of the collapse has been reported as marginal, having spared the parochial church^7^, or as catastrophic, having entrained and drowned the entire antique settlement of San Cristóbal-Kaj-Koj ^11^. The perfect continuity of the sedimentation during that time does not support an earthquake at that time and definitively rules out a catastrophic formation of the west sub-basin of the lake during the past twelve centuries.

In addition to the Motagua-Polochic system, the most important source of earthquake in Guatemala is the Cocos subduction zone. It has produced several major (M > 7) subduction earthquakes during the 20^th^ Century ^12^, but because of strong crustal attenuation inward of the subduction zone ^13^, earthquakes intensity never reached MMI ≥ VI at Lake Chichój. There is no evidence for significantly larger subduction earthquakes during previous centuries, which could have been felt with higher intensities at the lake ^12-15^. We assume therefore that all seismites recorded in the Chichój Lake sediments were produced by earthquakes generated on major and minor faults of the North American-Caribbean plate boundary.

**References**

1 Burkart, B. Offset across the Polochic fault of Guatemala and Chiapas, Mexico. *Geology* **6**, 328-332 (1978).

2 Authemayou, C. *et al.* Quaternary seismo-tectonic activity of the Polochic Fault, Guatemala. *Journal of Geophysical Research-Solid Earth* **117**, doi:10.1029/2012jb009444 (2012).

3 Brocard, G. *et al.* Reorganization of a deeply incised drainage: role of deformation, sedimentation and groundwater flow. *Basin Research* **23**, 631-651, doi:10.1111/j.1365-2117.2011.00510.x (2011).

4 Rose, W. I., Newhall, C. G., Bornhorst, T. J. & Self, S. Quaternary silicic pyroclastic deposits of atitlan caldera, Guatemala. *Journal of Volcanology and Geothermal Research* **33**, 57-80, doi:10.1016/0377-0273(87)90054-0 (1987).

5 Drexler, J. W., Rose, W., Sparks, R. & Ledbetter, M. The Los Chocoyos ash, Guatemala: a major stratigraphic marker in Middle America and in three ocean basins. *Quaternary Research* **13**, 327-345 (1980).

6 Brocard, G. *et al.* The recording of floods and earthquakes in Lake Chichój, Guatemala during the twentieth century. *Journal of Paleolimnology* **52**, 155-169, doi:10.1007/s10933-014-9784-4 (2014).

7 White, R. A. Catalog of historic seismicity in the vicinity of the Chixóy-Polochic and Motagua faults, Guatemala. *U.S. Geological Survey Open-File Report* **84-88**, 26 (1984).

8 White, R. A. The Guatemala earthquake of 1816 on the Chixóy-Polochic fault. *Bulletin of the Seismological Society of America* **75**, 455-473 (1985).

9 Guzman-Speziale, M. Beyond the Motagua and Polochic faults: Active strike-slip faulting along the Western North America-Caribbean plate boundary zone. *Tectonophysics* **496**, 17-27, doi:10.1016/j.tecto.2010.10.002 (2010).

10 Viana, F., Gallego, L. & Cadena, G. Relación de la provincia de la Verapaz, hecha por los religiosos de Santo Domingo de Cobán, Guatemala (1574). *An Soc Geog Hist Guatemala* **28**, 18-31 (1955).

11 Gage, T. *A new survey of the West-Indies: or the English American his Travel by Sea and Land*. (A. Clark, 1648).

12 White, R. A., Ligorría, J. P. & Cifuentes, I. L. Seismic history of the Middle America subduction zone along El Salvador, Guatemala, and Chiapas, Mexico: 1526–2000. *Geological Society of America Special Papers* **375**, 379-396 (2004).

13 Benito, M. B. *et al.* A new evaluation of seismic hazard for the Central America region. *Bulletin of the Seismological Society of America* **102**, 504-523 (2012).

14 Fernandez, M., Molina, E., Havskov, J. & Atakan, K. Tsunamis and tsunami hazards in Central America. *Natural Hazards* **22**, 91-116 (2000).

15 Montero, W. & Peraldo, G. Current knowledge on the Central America historical seismicity: an analysisof recent catalogues. *Annals of Geophysics* **47** (2004).
